# Comparison of gross tumor volume of primary oesophageal cancer based on contrast-enhanced three-dimensional, four-dimensional, and cone beam computed tomography

**DOI:** 10.18632/oncotarget.21520

**Published:** 2017-10-05

**Authors:** Chao-Yue Hu, Jian-Bin Li, Jin-Zhi Wang, Wei Wang, Feng-Xiang Li, Yan-Luan Guo

**Affiliations:** ^1^ School of Medicine and Life Sciences, University of Jinan-Shandong Academy of Medical Sciences, Jinan, Shandong Province, China; ^2^ Department of Radiation Oncology, Shandong Cancer Hospital Affiliated to Shandong University, Shandong Academy of Medical Sciences, Jinan, Shandong Province, China

**Keywords:** oesophageal cancer, three-dimensional computed tomography, four-dimensional computed tomography, cone beam computed tomography, gross tumor volume

## Abstract

**Background:**

To explore motion information included in 3DCT, 4DCT and CBCT by comparing volumetric and positional differences of GTV.

**Results:**

Independent of tumor location, significant differences were observed among volumes [IGTV_10_ > (IGTV_CBCT_ or IGTV_MIP_) > (GTV_3D_ or GTV_4D50_)]. The underestimations or overestimations between IGTV_10_ and IGTV_CBCT_ were larger than those between IGTV_10_ and IGTV_MIP_ (*p* < 0.001–0.011; *p* < 0.001–0.023). For upper oesophageal tumors, GTV_4D50_/IGTV_CBCT_ negatively correlated with motion vector (r = –0.756, *p* = 0.011). In AP direction, the centroid coordinates of IGTV_CBCT_ differed from GTV_3D_, GTV_4D50_, IGTV_MIP_ and IGTV_10_ (*p* = 0.006, 0.013, 0.038, and 0.010). For middle oesophageal tumors, IGTV_10_/IGTV_CBCT_ positively correlated with motion vector (r = 0.695, *p* = 0.006). The centroid coordinates of IGTV_CBCT_ differed from those of IGTV_10_ (*p* = 0.046) in AP direction. For distal oesophageal tumors, the centroid coordinates of IGTV_CBCT_ showed significant differences to those of IGTV_MIP_ (*p* = 0.042) in LR direction. For both middle and distal tumors, the degrees of associations of IGTV_10_ outside IGTV_CBCT_ significantly correlated with the motion vector (r = 0.540, *p* = 0.046; r = 0.678, *p* = 0.031).

**Materials and Methods:**

Thirty-four oesophageal cancer patients underwent 3DCT, 4DCT and CBCT. GTV_3D_, GTV_4D50_, internal GTV_MIP_ (IGTV_MIP_) and IGTV_CBCT_ were delineated on 3DCT, 4DCT_50_, 4DCT_MIP_ and CBCT. GTVs from 10 respiratory phases were combined to produce GTV_10_. Differences in volume, position for different targets, correlation between volume ratio and motion vector were evaluated. The motion vector was the spatial moving of the target centroid position.

**Conclusions:**

IGTV_CBCT_ encompasses more motion information than GTV_3D_ and GTV_4D50_ for upper oesophageal tumors, but slightly less than IGTV_10_ for middle and distal oesophageal tumors. IGTV_CBCT_ incorporated similar motion information to IGTV_MIP_. However, motion information encompassed in CBCT and MIP cannot replace each other.

## INTRODUCTION

Radiotherapy (RT) have been the mainstream technologies for the patients with inoperable oesophageal cancer. Different computed tomography (CT) images may be involved in the process of radiotherapy. Three-dimensional CT (3DCT) is a common positioning technology. In our study, the 3DCT images also known as “fast-CT” images were acquired in a short time. Although they only display the tumor during a certain moment of the breathing cycle [1], the temporal images acquired during 3DCT scanning encompass partial respiration-induced tumor motion information [2].

Oesophageal mobility can arise from peristalsis, respiratory motions, and cardiac action, with the predominant source of motion being respiratory [3]. Improved localization accuracy of the tumor can reduce the effect of radiation on the surrounding normal tissues and improve the therapeutic ratio. However, respiration-induced tumor motion can make target localization challenging [4–6]. Four-dimensional computed tomography (4DCT) is able to subdivide the CT datas acquired in a respiration cycle into a series of time-resolved 3D data [7, 8]. The advent of 4DCT has made the measure of respiration-induced motion of oesophageal mobility possible. Currently, during lung stereotactic body radiation therapy (SBRT), 4DCT is routinely incorporated into the treatment planning to include information regarding not only the range of tumor motion but also the different spatial tumor positions [9]. Regarding the definition of internal target volumes (ITV) according to the International Commission on Radiation Units and Measurements report 62, the internal gross tumor volume (IGTV) incorporating the intrafraction motion of the GTV has been adopted in many studies [2, 10]. In our present study, the relationships of IGTVs (IGTV_10_ combined from ten 4DCT phases and IGTV_MIP_ and IGTV_CBCT_) were analysed to explore the motion information included in IGTVs.

Prior to therapy delivery, it is crucial to localize the target volume using online imaging. Currently, cone beam CT (CBCT) is widely used for target verification, treatment planning modification, and image guidance during the delivery of radiation [11, 12]. In terms of online target verification and correction techniques, CBCT can show motion displacement of the GTV in three directions by fusing CBCT images with treatment planning CT scans. And CBCT scans are acquired over a period of several respiratory cycles providing motion information. Recent studies which mainly concentrated on lung tumors have gradually begun to give careful attention to the motion information obtained by CBCT [13–15]. However, comparisons of target volumes for oesophageal cancer are limited in the literatures, therefore it is essential to investigate the variations in motion information between the target volumes derived from 3DCT, 4DCT, and CBCT images for oesophageal tumors.

## RESULTS

### Variations of volumes

The means and standard deviations (SD) of the volumes of GTV_3D_, GTV_4D50_, IGTV_MIP_, IGTV_10_, and IGTV_CBCT_ are listed in Table 1. For upper, middle, and distal oesophageal tumors, the variation rule of the target volumes were completely consistent. The IGTV_10_ was larger than the IGTV_MIP_ and IGTV_CBCT_ (*p* < 0.001 for both), whereas no significant differences were observed between IGTV_CBCT_ and IGTV_MIP_ (*p* = 0.344, 0.580, and 0.128, respectively). The volumes of IGTV_CBCT_ and IGTV_MIP_ were also significantly larger than those of GTV_3D_ or GTV_4D50_ (*p* < 0.001–0.014), whereas there were no statistically significant differences between the GTV_3D_ and GTV_4D50_ (*p* = 0.691, 0.187, and 0.763, respectively).

**Table 1:** The volume of GTV_3D_ , GTV_4D50_, IGTV_MIP_, IGTV_10_ and IGTV_CBCT_ (Mean ± SD , cm^3^)

volume	GTV_3D_	GTV_4D50_	IGTV_MIP_	IGTV_10_	IGTV_CBCT_
upper	11.11 ± 2.86	11.18 ± 2.75	12.63 ± 2.93	13.80 ± 3.20	12.77 ± 3.12
middle	27.30 ± 12.46	27.74 ± 12.94	29.22 ± 12.94	33.52 ± 14.75	29.40 ± 12.70
distal	21.69 ± 11.88	21.78 ± 11.93	22.97 ± 12.17	27.86 ± 13.62	23.89 ± 11.99

The volume ratios of GTV_3D_, GTV_4D50_, IGTV_MIP_, and IGTV_10_ to IGTV_CBCT_ are listed in Table 2. For upper oesophageal tumors, the volume ratios of GTV_4D50_/IGTV_CBCT_ showed a significant correlation to the 3D direction motion vector (r = –0.756, *p* = 0.011). However, GTV_3D_/IGTV_CBCT_, IGTV_MIP_/IGTV_CBCT_, and IGTV_10_/IGTV_CBCT_ demonstrated no significant correlations to the 3D direction motion vector (*p* = 0.073–0.439). For middle oesophageal tumors, IGTV_10_/IGTV_CBCT_ showed a positive significant correlation to the 3D direction motion vector (r = 0.695, *p* = 0.006), whereas GTV_3D_/IGTV_CBCT_, GTV_4D50_/IGTV_CBCT_, and IGTV_MIP_/IGTV_CBCT_ did not (*p* = 0.091–0.762). For distal oesophageal tumors, no significant correlations were observed between the 3D direction motion vector and the volume ratios of GTV_3D_, GTV_4D50_, IGTV_MIP_, and IGTV_10_ to IGTV_CBCT_ (*p* = 0.131–0.921).

**Table 2:** The volume ratios of GTV_3D_, GTV_4D50_, IGTV_MIP_ and IGTV_10_ to IGTV_CBCT_ (Mean ± SD)

	GTV_3D_/IGTV_CBCT_	GTV_4D50_/IGTV_CBCT_	IGTV_MIP_/IGTV_CBCT_	IGTV_10_/IGTV_CBCT_
upper	0.87 ± 0.03	0.88 ± 0.05	0.99 ± 0.04	1.08 ± 0.03
middle	0.92 ± 0.04	0.93 ± 0.05	0.99 ± 0.04	1.14 ± 0.07
distal	0.87 ± 0.11	0.89 ± 0.09	0.94 ± 0.10	1.18 ± 0.07

### Variations of the target centroid position

The COM coordinates for all GTV or IGTV could be obtained through the TPS. For upper oesophageal tumors, in the AP direction, the centroid coordinates of IGTV_CBCT_ showed significant differences compared to those of GTV_3D_, GTV_4D50_, IGTV_MIP_, and IGTV_10_ (*p* = 0.006, 0.013, 0.038, and 0.010, respectively), whereas the target centroid positions at other directions showed no significant differences. For tumors located in the middle thoracic oesophagus, in the AP direction, there were significant differences of the centroid coordinates between IGTV_CBCT_ and GTV_3D_ (*p* = 0.008), and between IGTV_CBCT_ and IGTV_10_ (*p* = 0.046). Further, a significant difference was observed between the centroid position of IGTV_CBCT_ and GTV_4D50_ (*p* = 0.027) in the CC direction. For distal oesophageal tumors, variations in the centroid shift of IGTV_CBCT_ and GTV_3D_, IGTV_CBCT_ and GTV_4D50_, and IGTV_CBCT_ and IGTV_MIP_ were found only in the LR direction (*p* = 0.024, 0.017, and 0.042, respectively).

### Differences in the inclusion relation

The mean volumetric overestimations or underestimations for GTV_3D_, IGTV_MIP_, and IGTV_CBCT_ compared to the IGTV_10_ are listed in Table 3. Irrespective of the tumor location, the volumetric underestimation percentages of IGTV_10_ outside IGTV_CBCT_ were larger than those of IGTV_10_ outside IGTV_MIP_ (*p* < 0.001–0.011), whereas no significant differences were observed between IGTV_10_ outside IGTV_CBCT_ and IGTV_10_ outside GTV_3D_ (*p* = 0.357–0.943). The mean volumetric overestimations for IGTV_CBCT_ compared with IGTV_10_ were larger than those of IGTV_MIP_ compared with IGTV_10_ (*p* < 0.001–0.023). Similar results were found between IGTV_CBCT_ outside IGTV_10_ and GTV_3D_ outside IGTV_10_ (*p* < 0.001). For tumors located in the middle and distal thoracic oesophagus, the degrees of associations of IGTV_10_ outside IGTV_CBCT_ demonstrated significant correlations to the 3D direction motion vector (r = 0.540, *p* = 0.046; r = 0.678, *p* = 0.031).

**Table 3:** The volume characteristics for GTV_3D_, IGTV_MIP_ and IGTV_CBCT_ compared with IGTV_10_ (Mean ± SD, %)

	GTV_3D_ outside IGTV_10_	IGTV_10_ outside GTV_3D_	IGTV_MIP_ outside IGTV_10_	IGTV_10_ outside IGTV_MIP_	IGTV_CBCT_ outside IGTV_10_	IGTV_10_ outside IGTV_CBCT_
upper	2.80 ± 1.69	22.10 ± 3.64	5.20 ± 3.01	13.10 ± 2.69	16.00 ± 4.35	22.20 ± 3.82
middle	3.36 ± 2.90	21.60 ± 5.24	2.43 ± 2.06	14.79 ± 3.49	11.93 ± 4.	22.14 ± 2.63
distal	6.20 ± 7.65	29.70 ± 12.27	1.20 ± 0.42	20.70 ± 7.51	14.20 ± 6.41	26.90 ± 5.92

Abbreviations: GTV_3D_, the gross tumor volumes of three-dimensional computed tomography; GTV_4D50_, the gross tumor volumes of the end expiration phase of four-dimensional computed tomography; IGTV_MIP_, the internal gross tumor volumes of the maximum intensity projection of four-dimensional computed tomography; IGTV_10_, the union of gross tumor volumes from all ten phase images of four-dimensional computed tomography; IGTV_CBCT_, the internal gross tumor volumes of cone beam computed tomography.

### Differences in the MI

The mean values of the MIs between GTV_3D_ and IGTV_CBCT_, GTV_4D50_ and IGTV_CBCT_, IGTV_MIP_ and IGTV_CBCT_, and IGTV_10_ and IGTV_CBCT_ are listed in Table 4. The mean MIs ranged from 0.65 to 0.72. The MI values of IGTV_MIP_ relative to IGTV_CBCT_ were similar among the upper, middle, and distal thoracic oesophageal tumors (*p* = 0.235–0.863).

**Table 4:** The MIs between between GTV_3D_ and IGTV_CBCT_, GTV_4D50_ and IGTV_CBCT_, IGTV_MIP_ and IGTV_CBCT_, and IGTV_10_ and IGTV_CBCT_ (Mean ± SD)

	GTV_3D_-IGTV_CBCT_	GTV4_D50_-IGTV_CBCT_	IGTV_MIP_-IGTV_CBCT_	IGTV_10_-IGTV_CBCT_
upper	0.68 ± 0.06	0.65 ± 0.05	0.69 ± 0.06	0.68 ± 0.06
middle	0.72 ± 0.05	0.68 ± 0.04	0.71 ± 0.03	0.70 ± 0.03
distal	0.68 ± 0.06	0.67 ± 0.07	0.66 ± 0.09	0.65 ± 0.08

Abbreviations: GTV_3D_, the gross tumor volumes of three-dimensional computed tomography; GTV_4D50_, the gross tumor volumes of the end expiration phase of four-dimensional computed tomography; IGTV_MIP_, the internal gross tumor volumes of the maximum intensity projection of four-dimensional computed tomography; IGTV_10_, the union of gross tumor volumes from all ten phase images of four-dimensional computed tomography; IGTV_CBCT_, the internal gross tumor volumes of cone beam computed tomography.

## DISCUSSION

Target volume definition is a crucial step during the process of precise radiotherapy. The key to delineate GTV of oesophageal tumor is to define the boundaries of the primary tumor. The boundaries between the oesophagus and peripheral tissues such as the ventricles or descending aorta may be blurred on non-contrast enhanced CT images. Consequently, those problem will cause inaccuracies in the GTV delineation or IGTV construction. However, CE-CT scans can enhance the tumor margin between peripheral tissues, making it easy to determine the range of the tumor. Theoretically, in our study, intravenous contrast agents were used to acquired CE-CT images to improve the margin contrast between tissues and reduce the delineation uncertainty. Few studies have focused on the comparison of target volumes for oesophageal cancer using different CT imaging modalities. Therefore, we investigate the variations in motion information among the target volumes derived from 3DCT, 4DCT, and CBCT images for oesophageal tumors.

4DCT images, which is a novel method of portraying tumor motion, can provide the measure of breathing-induced tumor motion of the internal anatomy of the patient [16]. Previous studies considered that the end expiration phase was the most stable phase [17, 18], and GTV_4D50_ has been considered the closest measurement to the actual tumor size [19, 20]. Accordingly, the motion information encompassed in GTV_4D50_ is considered the lowest. In our data, we demonstrated that GTV_4D50_/IGTV_CBCT_ showed a significant inverse correlation to motion vector for upper oesophageal tumors, indicating an increase in the variation of the IGTV_CBCT_ size with the increasing tumor motion amplitude. This might be due to the GTV_4D50_ was considered the closest measurement to the actual tumor size, and would thus reflect the actual oesophageal tumor size [20]. In addition, oesophageal mobility can arise from peristalsis and respiratory and cardiac actions. The upper oesophagus passes through the sternal notch and above the left atrium, along with numerous other structures, and is therefore generally very well fixed. Hence, the upper oesophagus has been reported to be the least affected by cardiac action (left atrium) and respiratory motion, with the lowest motion amplitudes (0.16, 0.14, and 0.29 cm in the LR, AP, and CC directions, respectively) [10]. Consequently, GTV_4D50_ could reflect the actual oesophageal tumor size. However, the CBCT scans are acquired over a period of several respiratory cycles providing sufficient information on respiration motion. Therefore, the motion information encompassed in the IGTV_CBCT_ can be accurately reflected by comparing the variation in volume sizes between IGTV_CBCT_ and GTV_4D50_. In this study, the volume ratio correlated well with the motion vector, suggesting that IGTV_CBCT_ incorporates much more motion information than GTV_4D50_ for upper oesophageal tumors.

At present, CE-3DCT was widely used for the positioning of oesophageal cancer as most patients can tolerate it. Generally speaking, margins derived from population-based or site-specific tumor motion information of 4DCT are used to guide the expansion from GTV based on 3DCT to IGTV. However, the crucial premise is that 3DCT does not encompass much more motion information than the GTV from a single phase. Herein, we demonstrated that the ratios of GTV_3D_/GTV_4D50_ for the upper, middle, and distal oesophagus approached 1, similar to what has been reported for lung cancer [2, 20]. This result suggests that GTV_3D_ encompasses similar motion information as GTV_4D50_. Consequently, we considered that IGTV_CBCT_ incorporates much more respiration motion information than GTV_3D._ Given that the centroid coordinates between GTV_4D50_ and GTV_3D_ showed no significant difference in any of the three directions (*p* = 0.204, 0.149, and 0.505, respectively), and the relative high inclusion relations of GTV_3D_ in GTV_4D50_ and GTV_4D50_ in GTV_3D_ (0.88 and 0.87, respectively), which demonstrated no significant difference for the whole group (t = 1.525, *p* = 0.137), 3DCT may be regarded as an alternative when 4DCT images are not or cannot be acquired.

During the course radiotherapy, 3DCT simulation scans were routinely used for patient's positioning, and CBCT were widely used for target verification and image guidance during the delivery of radiation. As technology advances, the image resolution and scanning range of CBCT has improved. The applications of CBCT are further explored. 3DCT scanning repositioning are usually needful to define the patient's position repeatability and the necessity of modifying the target during the course of radiotherapy. So we explore the matching of the motion information included in 3DCT and CBCT to realize whether we could utilize CBCT instead of 3DCT to modify the treatment planning. Whether we could delineate IGTV_CBCT_ on CBCT images acquired after times of radiotherapy, and then map IGTV_CBCT_ to first positioned 3DCT directly to modify the treatment planning without the course of 3DCT scanning repositioning, provided that: a) the two target volumes match very well delineated on CBCT images acquired at the first treatment fraction and first positioned 3DCT images; b) the patient's position repeatability was very perfect during the course of radiotherapy; c) the tumor was obviously shrinked during the course of radiotherapy. We analysed the inclusion relations between GTV_3D_ and IGTV_10_, and between IGTV_CBCT_ and IGTV_10_. IGTV_10_ has been reported to provide the best overall representation of the ‘true’ moving GTV and incorporates the whole tumor respiration motion information envelope throughout the entire breathing cycle, though its delineation is the most time-consuming [10, 21, 22]. Therefore, we regarded IGTV_10_ as the reference to reflect the differences in motion information contained in the target volumes on different CT modalities. However, in the part of results we found that the proportion of the volumetric overestimations for IGTV_CBCT_ outside IGTV_10_ were larger than those for GTV_3D_ outside IGTV_10_. The result did not meet the first premise. Therefore, 3DCT simulation scans are needed for repositioning during the course of radiotherapy.

From the results of the present study, we found no significant difference among the IGTV_MIP_/IGTV_CBCT_ for upper, middle, and distal oesophageal tumors (F = 2.043, *p* = 0.147). Meanwhile, no good correlation was observed between the motion vector and IGTV_MIP_/IGTV_CBCT_, irrespective of where the tumor was located (r = –0.301, *p* = 0.398; r = 0.458, *p* = 0.099; and r = –0.113, *p* = 0.755, respectively). These results indicated that the tumor motion amplitude had no effect on the variation in IGTV_MIP_/IGTV_C_BCT and that the changes in IGTV_CBCT_ and IGTV_MIP_ may have a similar magnitude. Therefore, this suggests that the motion information encompassed in IGTV_CBCT_ was parallel to that of IGTV_MIP_.

Independent of the tumor location, the percentages of volumetric underestimations or overestimations between IGTV_10_ and IGTV_CBCT_ were larger than those of IGTV_10_ and IGTV_MIP_ in the present study. The MIs for IGTV_MIP_ relative to IGTV_CBCT_ were only 0.69, 0.71, and 0.66 for upper, middle and distal thoracic oesophageal tumors, respectively. The main cause of this phenomenon may be the variations in the target centroid position between IGTV_MIP_ and IGTV_CBCT_. Although for middle and distal oesophageal tumors, the predominant source of motion is respiratory [3]. As a tube-shaped and non-rigid organ in the mediastinum, in addition to peristalsis and respiration-induced movement, motion of the heart and aorta resulting from the cardiac cycle can also result in motion and volume deformation of the oesophagus [3, 23]. Accordingly, respiration-induced movement, and the squashing and stretching of the heart to the esophageal tumors might be factors responsible for the differences in the COM between IGTV_MIP_ and IGTV_CBCT_ in the AP and LR directions. Especially, for middle and distal oesophageal cancer, the effects of the variations in the position and volume of the heart on oesophageal targets cannot be ignored [24]. Of note, similar findings have been observed in lung cancer, as Seppenwoold et al. [25] reported that the target was affected by cardiac action more obviously in the LR direction when the lung tumors were located near the heart. Thus, IGTV_CBCT_ and IGTV_MIP_ encompass similar respiration motion information; however, due to the spatial mismatch of IGTV_CBCT_ and IGTV_MIP_, the target motion information encompassed in CBCT and MIP images cannot replace each other.

The present study showed that the volume of IGTV_10_ was larger than IGTV_CBCT_ and, simultaneously, that IGTV_10_/IGTV_CBCT_ correlated well with the motion vector. These findings demonstrate that the change in IGTV_10_ was more obvious than the change in IGTV_CBCT_ along with the increase in the tumor motion amplitude. In this study, the volume ratio correlated well with the motion vector, suggesting that IGTV_10_ incorporates much more motion information than IGTV_CBCT_. When comparing IGTV sizes, we found that if IGTV_10_ was chosen as the reference for the standard volume, 22.20%, 22.14%, and 26.90% of IGTV_10_ volume tumor tissues would not receive irradiation, whereas 16.00%, 11.93%, and 14.20% of the volume IGTV_CBCT_ normal tissues would be inevitably irradiated for upper, middle, and distal oesophageal tumors, respectively. Furthermore, our study showed the correlation of IGTV_10_ outside IGTV_CBCT_ and the spatial motion vector. This result showed the proportion of IGTV_10_ outside IGTV_CBCT_ increased as the tumor motion increased. It indicated that the larger the motion amplitude of the tumors, the more motion information of IGTV_CBCT_ would be missed compared to IGTV_10_. The MI and inclusion relation reflect the translation, deformation, volume change, or rotation of the two selected volumes, which in turn affect the volumetric size, shape, and spatial position [26]. The variations of the target centroid position between IGTV_10_ and IGTV_CBCT_ may be a factor leading to the volumetric mismatch.

For upper oesophageal tumors, in the AP direction, the centroid coordinates of IGTV_CBCT_ differed from those of IGTV_10_ in the present study. As the upper oesophagus is generally very well fixed by numerous adjacent structures, it is the least affected by cardiac action and respiratory motion, with the least motion amplitude. Oesophageal mobility can arise from peristalsis, and attention should be paid to the day-to-day oesophageal peristalsis [12], as oesophageal intrafractional tumor position variation during irradiation delivery is one of the geometrical uncertainties that may affect the target centroid position. Oesophageal peristalsis in the AP direction might contribute to spatial position differences, which consequently leads to the differences in the quantity of motion information incorporated in IGTV_CBCT_ and IGTV_10_. Furthermore, setup errors might have also contributed to the observed difference. Yamashita et al. [12] reported that the setup error was 4 mm (maximum, 11 mm) in the longitudinal direction, 2 mm (maximum, 8 mm) in the lateral direction, and 4 mm (maximum, 13 mm) in the vertical direction.

Other factors that likely contributed to the differences in spatial mismatch and volume size between the two target volumes should also be highlighted. First, free breathing CBCT image reconstruction makes use of every pixel in each projection, resulting in an averaging of the CT numbers across the image set. Accordingly, this image is meant to represent the time-averaged position of the target and results in a CT number averaging effect [15, 27]. However, 4DCT images were a series of time-resolved 3D data. Due to the different image principle of 4DCT and CBCT, it may be one factor to lead the difference of the volume size and volumetric mismatch between two target volumes on different CT images. In addition, variations in the breathing pattern may be another potential reason for the observed differences during the acquisition of 3DCT, 4DCT, and CBCT scans. Clements et al. [15] reported that the sinusoidal patterns represented the ideal clinical scenario. However, in the present study, all CT image acquisitions were acquired during free breathing, without any breathing control, and this is bound to the variations of the target centroid position and volume size , as well as to the low MI between the two selected volumes.

There were some limitations in the present study. Most importantly, it should be noted that intraobserver target delineation error might reduce the accuracy of GTVs delineated on distinct patterns of CT images. To minimize systematic delineation uncertainty, the contouring was performed by a single physician with more than 5 years of experience using a unified standard, despite the fact that contouring the target volume on 10 different phases increases the workload and is time-consuming. Additionally, it is impossible to avoid the impact of registration errors. Boswell et al. [28] reported that the repositioning accuracy of automatic registration in a helical tomotherapy system was sub-millimetre. However, errors in alignment and patient setup displacements still exist, and it is possible that such errors, albeit minor, may have caused variations in the target centroid position and resulted in the low matching between the two target volumes. Nevertheless, despite the limitations in this study, our results can reflect the amount of respiration motion information encompassed in different CT modalities.

## MATERIALS AND METHODS

### Patient selection and characteristics

Thirty-four patients (19 men and 15 women) with pathologically confirmed oesophageal cancer, scheduled to undergo radiotherapy with 3D-CRT or IMRT, were enrolled between August 2014 and December 2015. The median age was 71 years (range, 41–83 years). Among the 34 patients, 33 and 1 were diagnosed with squamous cell carcinoma and undifferentiated carcinoma, respectively. The primary tumors were located in the upper, middle, and distal oesophagus in 10, 14, and 10 patients, respectively, based on the 7th edition of Esophageal Cancer Staging published in 2009 from the American Joint Committee on Cancer (AJCC) and Union for International Cancer Control (UICC). No patient had been previously treated with thoracic radiotherapy and no patient had poor pulmonary function.

Written informed consent was obtained from all patients before the treatment was initiated. The study was approved by the Institutional Review Board (Shandong Cancer Hospital Affiliated to Shandong University Ethics Committee).

### CT data acquisition

All patients were in the vacuum bags while being scanned. After laser alignment, contrast enhanced (CE)-3DCT and CE-4DCT were performed during free breathing using a 16-slice CT scanner (Philips Brilliance Bores CT Inc., Cleveland, OH, USA). The standard acquisition parameters of the CE-3DCT were 120 kV and 200 mA. A total of 45 mL of iodinated contrast medium were infused at a rate of 1.5 mL/s. The CE-3DCT scans were produced per gantry rotation (1 s) and interval (1.8 s) between rotations. The slice thickness of the 3DCT scan was 3 mm. When the CE-3DCT was finished, the CE-4DCT scan was sequentially initiated. The standard acquisition parameters of the CE-4DCT were 120 kV and 400 mA. A total of 55 mL of iodinated contrast medium were infused continuously at a rate of 1.0 mL/s. The CE-4DCT scan was acquired in helical mode with the scanning pitch ranging between 0.09 and 0.15. During the CE-4DCT image acquisition, the respiratory signal was recorded with the Varian real-time position management (RPM) system (Varian Medical Systems, Palo Alto, CA, USA) by tracking the trajectory of the infrared markers placed on the epigastric region of the patient's abdomen. The resultant signal was sent to the scanner to label a time tag on each image. GE Advantage 4D software (GE Healthcare, Waukesha, WI, USA) was used to sort the reconstructed 4DCT images into 10 respiratory phases according to the phase of the breathing signal based on these tags labelled as 0–90%. Phase 0% denoted the maximum end inspiration, and phase 50% denoted the maximum end expiration. The maximum intensity projection (MIP) images were created from the raw 4DCT data. In the MIP images, each pixel was assigned the highest density value that occurred, taking into account all 10 respiratory phases.

Prior to the first treatment fraction, CE-CBCT scans were acquired using a kilovoltage CBCT scanner (Varian Medical Systems, Palo Alto, CA, USA) with the patient in the treatment position. The patients were aligned according to the skin tattoos by using the in-room laser system. The standard acquisition parameters were 120 kV and 1000 mA. The iodinated contrast medium was infused at a rate of 1.8 mL/s. The scan time was approximately one minute. The Eclipse system included software using a mutual information algorithm for automatic registration from the CE-4DCT and CE-CBCT to CE-3DCT. Finally, all images were transferred to the Eclipse treatment planning system (TPS) (Eclipse 8.6; Varian Medical Systems, Palo Alto, CA) for structure delineation and treatment planning. In our study, the 3DCT images were used for target delineation and treatment planning. 4DCT images were used for target delineation. The CBCT was used for target delineation and verification.

### GTV delineation

**Figure 1:**
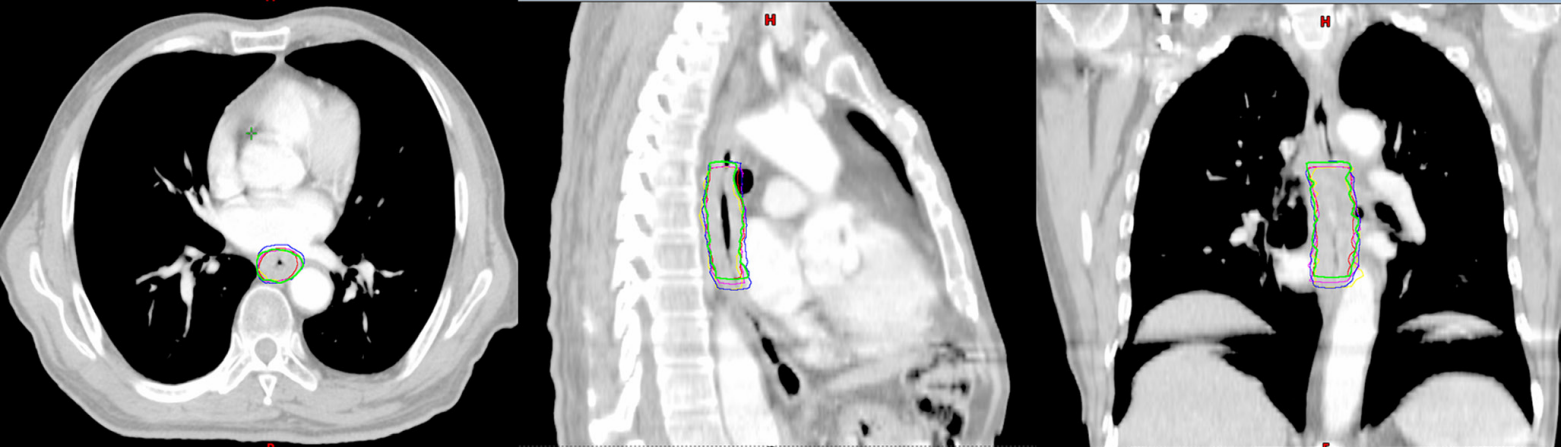
Gross target volume (GTV) or internal GTV (IGTV) formation in one patient: (green), GTV3D; (red), GTV_4D50_; (purple) IGTV_MIP_; (blue) IGTV_10_; (yellow) IGTV_CBCT_ The figures are the axial, sagittal and coronal view of one patient exhibiting the GTV or IGTVs.

In all cases, the GTV and/or IGTV contouring were completed by the same clinician, with more than 5 years of experience performing target volume delineation, using the mediastinal window setting. The GTV and/or IGTV for each patient were delineated as follows: (1) the GTV_3D_ and GTV_4D50_ were delineated on CE-3DCT and CE-4DCT_50_ images (the end expiration phase of CE-4DCT), respectively; (2) the IGTV_MIP_ and IGTV_CBCT_ were delineated separately on the CE-4DCT_MIP_ (the maximum intensity projection of CE-4DCT) and CE-CBCT datasets, respectively; and (3) the GTVs on each of the 10 respiratory phases of the CE-4DCT images were delineated and combined to produce IGTV_10_ (Figure 1).

### Tumor motion

The centre of mass (COM) coordinates represent the target centroid position. The target centroid position was reflected by the COM coordinates. The COM coordinates for all GTV or IGTV could be obtained through the TPS. The intrafractional displacements of the COM in the *x* (left-right [LR]), *y* (anterior-posterior [AP]), and *z* (cranial-caudal [CC]) directions throughout 10 phases of 4DCT could also be obtained. The maximum tumor displacement in the LR, AP, and CC directions throughout 10 phases of 4DCT were calculated as Δ*x*, Δ*y*, and Δ*z*, respectively. Subsequently, the 3D motion vector of the COM from the 4DCT was calculated according to the following formula:

$$\text{Vector}=\sqrt{\text{D}x^{2}+\text{D}y^{2}+\text{D}z^{2}}$$

### Volume comparisons

The volume, inclusion relation, and matching index (MI) were compared among the GTV (GTV_3D_ and GTV_4D50_) and IGTV (IGTV_MIP_, IGTV_10_, and IGTV_CBCT_). The percentage of A not included in B [Per (A not in B)] is used to indicate the inclusion relation between two volumes. The target A outside B was represented by Per (A not in B). Assumed volume B was reference for the standard volume irradiated. If the treatment planning was based on volume A, there would be Per (B not in A) of volume B missing irradiation which means the volumetric underestimation and Per (A not in B) of volume A being irradiated unnecessarily which means the volumetric overestimation. The formula is as follows: Per (A not in B) = 1- A∩B/A [21].

The MI was defined as the ratio of the intersection of volume A with volume B divided by the union of A and B, as follows: MI = A∩B/A∪B [26]. The MI indicated translation, deformation, volume change, or rotation of the two selected volumes. The ideal value of the MI is 1; upon any change in size, shape, position, or orientation, the value of MI would be < 1.

### Statistical analyses

Statistical analyses were performed using SPSS software (version 19.0; SPSS Inc., Chicago, IL, USA). For all parameters, a normal distribution test (Q-Q plot) was performed. The paired sample t test was used for comparisons of tumor position, volumetric size, MI, and inclusion relation. The degree of associations between continuous variables and the 3D motion vector according to 4DCT was calculated using Pearson's test. For all analyses, a *p* value < 0.05 was regarded as significant.

## CONCLUSIONS

Our findings indicate that the CBCT images incorporated much more respiration motion information than 3DCT images and the end expiration phase of 4DCT images for upper oesophageal tumors, but less than that of 10 respiratory phases of the 4DCT datasets for middle and distal oesophageal tumor. Simultaneously, CBCT images incorporated similar respiration motion information to MIP images. Nevertheless, the target motion information encompassed in CBCT and MIP images cannot replace each other.
